# Response to comment on “Humic acid-dependent respiratory growth of *Methanosarcina acetivorans* involves pyrroloquinoline quinone” by Yuanxu Song *et al.*

**DOI:** 10.1093/ismejo/wrae019

**Published:** 2024-01-31

**Authors:** Yuanxu Song, Rui Huang, Ling Li, Mingyu Wang, Shuguang Wang, James G Ferry, Zhen Yan

**Affiliations:** Shandong Key Laboratory of Water Pollution Control and Resource Reuse, School of Environmental Science and Engineering, Shandong University, Qingdao 266237, China; Shandong Key Laboratory of Water Pollution Control and Resource Reuse, School of Environmental Science and Engineering, Shandong University, Qingdao 266237, China; State Key Laboratory of Microbial Technology, Microbial Technology Institute, Shandong University, Qingdao, Shandong 266237, China; State Key Laboratory of Microbial Technology, Microbial Technology Institute, Shandong University, Qingdao, Shandong 266237, China; Shandong Key Laboratory of Water Pollution Control and Resource Reuse, School of Environmental Science and Engineering, Shandong University, Qingdao 266237, China; Department of Biochemistry and Molecular Biology, Pennsylvania State University, University Park, PA 16801, United States; Shandong Key Laboratory of Water Pollution Control and Resource Reuse, School of Environmental Science and Engineering, Shandong University, Qingdao 266237, China

**Keywords:** Respiratory growth, Methanogenesis, Electron transfer, Humic acid

Microbial respiration by humic substances reduction is of significance to organic matter decomposition and biogeochemical cycling of key elements in anoxic environments. In a recent study, we determined that a species of methanogenic archaea, *Methanosarcina acetivorans*, which normally acquires energy by fermentative methanogenesis, is also capable of undergoing simultaneous fermentation and humic acid (HA) respiration for energy conservation [1]. The results of this study clearly demonstrated that the presence of HA enhanced the growth of *M. acetivorans* above that attributed to methanogenesis when utilizing the energy sources methanol or acetate, and that a cluster of genes annotated as cell-surface pyrroloquinoline quinone-binding proteins were significantly upregulated in HA-respiring cells. However, Lovley and Holmes argued that HA reduction during growth of *M. acetivorans* was negligible, so the growth phenotype of the organism and the transcriptomic regulation that occurred in the presence of HA had little relevance to HA respiration [2].

This assertion by Lovley and Holmes is largely based on a solitary analysis of the stoichiometry of methane produced from methanol at the end of the stationary phase of growth [2]. These authors suggested that in both the presence and absence of HA similar amounts of methane were produced from the same amount of methanol, which precludes diversion of reductant to HA for respiratory growth. However, as described below, a relatively small reduction in total methane is expected from diversion of electrons to HA, which complicates conclusions based on stoichiometry alone, especially at the end of growth. Notably, the levels of methanol at the end of growth were at the extreme of detection, which precludes reliable measurements to predict stoichiometry. Here, we present analyses of pre-existing data prior to the stationary phase (Fig. 1), which counter the conclusions drawn by Lovley and Holmes [2].

**Figure 1:**
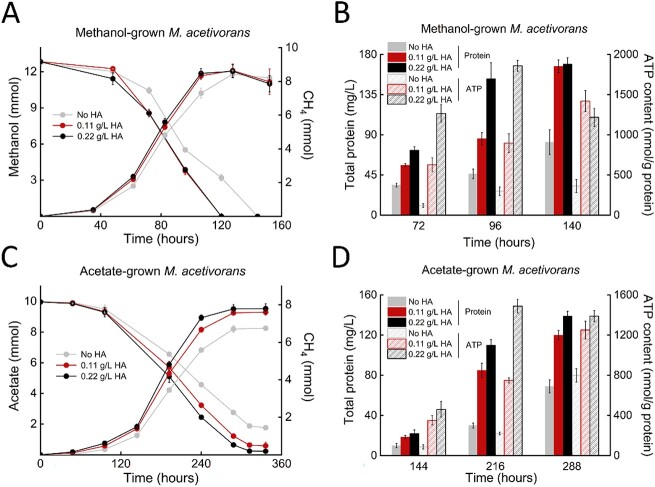
Growth parameters of *M. acetivorans* grown with or without HA. Time-dependent methane production and carbon source utilization of methanol-grown *M. acetivorans* (A) and acetate-grown *M. acetivorans* (C). Time-dependent accumulation of cellular protein and ATP of methanol-grown *M. acetivorans* (B) and acetate-grown *M. acetivorans* (D). This figure is a replicate of the Figs. 1A–1D in our publication [1] claimed by Lovley and Holmes [2].

For the methanol experiment, an interpolated 9.0 mmol was consumed in the absence of HA at ~115 h, whereas the same consumption of 9.0 mmol was measured for cultures with HA at ~100 h (Fig. 1A). Assuming 10% of consumed methanol is diverted for cell carbon, 8.1 mmol was metabolized for energy conservation, of which ~6.1 mmol (8.1 × 3/4) of methane is expected by fermentative methanogenesis for the HA-null cultures (4CH_3_OH = 2H_2_O + 3CH_4_ + CO_2_). Interpolation revealed 7.8 mmol of methane produced at ~115 h for the HA-null cultures, which indicates an overestimation. As described in the calculation section below, a 5-fold greater amount of energy conservation was calculated for HA respiration than for fermentative methanogenesis based on thermodynamic considerations. Figure 1B shows that respiration and fermentation contributed equally to growth. Thus, ~1.6 mmol (8.1 × 1/5) methanol was metabolized for respiration, reducing the amount of methane by 1.2 mmol (1.6 × 3/4) from the 6.1 mmol methane expected by fermentation alone. Assuming that the overestimation of methane applies equally, the difference in measured methane produced from 8.1 mmol methanol in the presence (7.0 mmol) and absence (7.8 mmol) of HA is consistent with the calculations. This trend is consistent with comparisons between the time points for consumption of 5.0 mmol methanol at ~85 h in the absence of HA and ~75 h in the presence of HA. These findings highlight the small differences in methane expected between cultures with and without added HA and the difficulty in discerning differences on a larger background of methane.

The validity of the assessment described above is striking when the same analyses are applied for growth with acetate. A measured 5.5 mmol of acetate was consumed in the absence of HA at ~240 h, whereas the same consumption of 5.5 mmol was measured for cultures with 0.22 g/L HA at ~200 h (Fig. 1C). Assuming that 10% of consumed acetate is diverted for cell carbon, 5.0 mmol was metabolized for energy conservation, of which 5.0 mmol of methane is expected by fermentative methanogenesis for the HA-null cultures (CH_3_COOH = CH_4_ + CO_2_). Interpolation revealed that 5.6 mmol of methane was produced at ~240 h for the HA-null cultures, which indicates an overestimation that likely also applies to HA-amended cultures. As described in the calculation section below, based on thermodynamic considerations a 12.7-fold greater amount of energy conservation was calculated for HA respiration than for fermentative methanogenesis. Figure 1D shows that respiration and fermentation contributed equally to growth. Thus, ~0.4 mmol (5 × 1/12.7) acetate was metabolized for respiration, a reduction of 0.4 mmol methane from the 5.0 mmol methane expected by fermentation alone. The difference in measured methane produced from 5.0 mmol acetate in the presence (5.3 mmol) and absence (5.6 mmol) of HA is consistent with the calculations. This result further highlights the small differences in methane expected between cultures with and without added HA and the difficulty in discerning differences on a larger background of methane. Nonetheless, the analyses presented here do not invalidate our conclusion that HA respiration contributed to growth, which Lovley and Holmes questioned based on analyses of methane produced at the end of growth. Indeed, the analyses presented here are consistent with our conclusions [2].

Moreover, other published accounts corroborate these analyses. For the well-recognized Fe(III)-dependent respiratory growth of *Methanosarcina* spp., Yang et al. reported that *Methanosarcina mazei* reduced extracellular Fe(III) during methanogenic growth with acetate, whereas no significant difference in total methane production was apparent between the group with added Fe(III) and the control group without Fe(III) [3]. However, by directly measuring Fe(III) reduction, Yang et al. calculated that only 1.7%–3.5% of the total electrons from substrate utilization were used for respiration. The 1.7%–3.5% of total electrons used for respiration, however, supported approximately 36%–46% of the cell growth, indicating that extracellular respiration is much more efficient than fermentative methanogenesis for energy conservation. Similar results were reported by Prakash et al., who observed no apparent differences in total methane between the Fe(III)-added group and the control group without Fe(III) when equal moles of acetate were consumed, and these authors attributed ~40% of the growth to Fe(III) respiration during acetate-dependent growth of *M. acetivorans* [4].

The question then arises as to how many electrons derived from the oxidation of methanol or acetate were diverted to reduce extracellular HA. Because HA has multiple types of redox functional groups, resulting in uncertainty regarding its reduction, and also because there is no documented method to directly quantify HA reduction, we did not discuss this point in our article. Lovley and Holmes calculated that only 0.01%–0.02% of substrate electrons were diverted extracellularly to reduce HA by using an electron-accepting capacity of 0.153 mmol of electrons/g HA, a value they previously reported in 1999 [5]. More than 10 years ago, the electron-accepting capacities of HA were generally determined chemically. Later, electrochemical methods were established to determine the electron-accepting capacities of HA, demonstrating that the chemical methods were very inaccurate [6]. Nevertheless, the electron-accepting capacities of HA determined by electrochemical methods vary significantly due to different redox states of HA under different electrochemical conditions. For example, in previous work the investigators searched a large amount of data on electron-accepting capacities of HA measured by electrochemical methods and found that the electron-accepting capacities varied from 0.11 to 2.66 mmol of electrons/g HA [6]. Before selecting one of the reported values for calculating the amounts of HA reduction in our work, clarification is needed regarding whether the functional groups that are involved in the reduction of HA during measurement of its electron-accepting capacity are consistent with the functional groups of the biologically reduced HA. Therefore, the electron-accepting capacity of HA reported by Lovley et al. in 1999 is not appropriate for use in this calculation [4].

We showed that HA enhancement of the growth of *M. acetivorans*, determined by monitoring the levels of protein and ATP, was above that attributable to methanogenesis, which indicates both respiration and fermentation. Lovley and Holmes argued that HA may interfere with the protein measurement, or that impurities in HA may serve as trace elements enhancing growth. However, we ruled out in a pretest the possibility that the added HA would interfere with protein measurement. In addition, none of the many previous publications on HA as an electron acceptor for microbial respiration have reported that the potential impurities in HA were used as nutrients for microbial growth.

Holmes et al. reported methanol-dependent respiratory growth of *M. acetivorans* with anthraquinone-2,6-disulfonate (AQDS) [6], a quinone-like artificial electron acceptor, and stated that the electron-accepting capacity of the AQDS they used was much more than that of the added HA in our work. Although AQDS is often used as an analog of HA, HA is much richer in redox functional groups in addition to the quinone group [6]. To understand the ecological role of HA in methanogens, it is important to use a natural electron acceptor instead of an artificial one. More importantly, consideration is needed as to whether the concentration level of the electron acceptor used makes environmental sense. Holmes et al. used 16 mM (6.6 g/L) of AQDS in their previously reported study, a concentration level that could not possibly be reached with environmental HA. By contrast, we used 0.11–0.22 g/L HA in our work, which is a level similar to previously reported concentrations of environmental HA [8, 9]. Thus, the HA-dependent respiratory growth of *M. acetivorans* we reported has environmental implications.

In addition to our report concerning HA-dependent respiratory growth of *M. acetivorans* published in the *ISME Journal*, we recently also reported that HA respiration drives methanotrophic growth of *M. acetivorans* [10]. In this investigation HA was shown to be an electron acceptor with methane as the sole carbon and energy source and electron donor, enabling considerable cell growth and respiratory energy conservation. In addition, the capability of HA to serve as a terminal electron acceptor for sustaining growth of the anaerobic methanotrophic archaea-2 clade that belongs to *Methanosarcinales* has also been documented [11, 12]. These reports further support our finding that *M. acetivorans* is capable of reducing HA, a process that plays an important role in the ecophysiology of methanogens.

**Calculation 1: standard Gibbs free energy of HA-dependent respiratory growth with methanol as a carbon source**

CH_3_OH + 2F_420_ + Fd + H_2_O → 2F_420_H_2_ + Fd^−2^ + 2H^+^ + CO_2_.

ΔG′ = −3.0 kJ [13].

2F_420_H_2_ + 2HA → 2HAH_2_ + 2F_420_.

ΔE′ = −360 [13] + 300 [14, 15] = 660 mV.

ΔG′ = –nFΔE′ = −4(96 485 J/mol/V) 0.660 = −254.7 kJ.

Fd^−2^ + 2H^+^ + HA → HAH_2_ + Fd.

ΔE′ = −407 [16] + 300 = 707 mV.

ΔG′ = −nFΔE°′ = −2(96 485 J/mol/V) 0.707 = −136.4 kJ.

**------------------------------------------------------------------------------------------------**

**Sum:** CH_3_OH + H_2_O + 3HA → 3HAH_2_ + CO_2_.

ΔG′ = − 3.0 kJ – 254.7 –136.4 = −394.1 kJ/CH_3_OH.

4CH_3_OH → CO_2_ + 3CH_4_ + 2H_2_O.

ΔG′ = −106.5 kJ/CH_4_ = −79.9 kJ/CH_3_OH [13].

Fold change: −394.1 kJ/−79.9 kJ = 4.9.

**Calculation 2: standard Gibbs free energy of HA-dependent respiratory growth with acetate as a carbon source**

CH_3_COOH + H_4_SPT + Fd + H_2_O → Fd^−2^ + 2H^+^ + CH_3_-H_4_SPT + CO_2_.

ΔG′ = +77.0 kJ [13].

CH_3_-H_4_SPT+2F_420_+Fd+H_2_O →2F_420_H_2_+Fd^−2^ + 2H^+^+ H_4_SPT + CO_2_.

ΔG′ = −6.3 kJ [13].

2F_420_H_2_ + 2HA → 2HAH_2_ + 2F_420_.

ΔE′ = −360 [13] + 300 [14, 15] = 660 mV.

ΔG′ = –nFΔE = −4(96 485 J/mol/V) 0.660 = −254.7 kJ.

2Fd^−2^ + 4H^+^ + 2HA → 2HAH_2_ + 2Fd.

ΔE′ = −407 [16] + 300 = 707 mV.

ΔG′ = -nFΔE°′ = −4(96 485 J/mol/V) 0.707 = −272.9 kJ.

**------------------------------------------------------------------------------------------------**

**Sum:** CH_3_COOH + 2H_2_O + 4HA → 4HAH_2_ + 2CO_2_.

ΔG′ = 77.0–6.3 – 254.7 – 272.9 = −456.9 kJ/CH_3_COOH.

CH_3_COOH → CH_4_ + CO_2_.

ΔG′ = −36.0 kJ/CH_3_COOH [13].

Fold change: −456.9 kJ/−36.0 kJ = 12.7.

## Data Availability

The authors acknowledge that no data were generated requiring deposit in publicly accessible databases.

## References

[1] Song YX , Huang R, Li L et al. Humic acid-dependent respiratory growth of Methanosarcina acetivorans involves pyrroloquinoline quinone. Isme J 2023;17:2103–11. 10.1038/s41396-023-01520-y. 37737251 PMC10579383

[2] Lovley DR , Holmes DE. Comment on “Humic acid-dependent respiratory growth of Methanosarcina acetivorans involves pyrroloquinoline quinone” byYuanxu song et al. Isme J 2024:wrae020. 10.1093/ismejo/wrae020. PMC10945356 38366259

[3] Yang Z , Lu Y. Coupling methanogenesis with iron reduction by acetotrophic Methanosarcina mazei zm-15. Environ Microbiol Rep 2022;14:804–11. 10.1111/1758-2229.13098. 35641250

[4] Prakash D , Chauhan SS, Ferry JG. Life on the thermodynamic edge: respiratory growth of an acetotrophic methanogen. Sci Adv 2019;5(8):eaaw9059. 10.1126/sciadv.aaw9059. PMC6703866 31457094

[5] Lovley DR , Blunt-Harris EL. Role of humic-bound iron as an electron transfer agent in dissimilatory Fe(III) reduction. Appl Environ Microb 1999;65:4252–4. 10.1128/AEM.65.9.4252-4254.1999. PMC99772 10473447

[6] Ou JJ , Wen JL, Tan WB et al. A data-driven approach for understanding the structure dependence of redox activity in humic substances. Environ Res 2023;219:115142. 10.1016/j.envres.2022.115142. 36566968

[7] Holmes DE , Ueki T, Tang HY et al. A membrane-bound cytochrome enables Methanosarcina acetivorans to conserve energy from extracellular electron transfer. MBio 2019;10:e00789–19. 10.1128/mBio.00789-19. 31431545 PMC6703419

[8] Filella M . Quantifying 'humics' in freshwaters: purpose and methods. Chem Ecol 2010;26:177–86. 10.1080/02757540.2010.494159.

[9] Philippe A , Schaumann GE. Interactions of dissolved organic matter with natural and engineered inorganic colloids: a review. Environ Sci Technol 2014;48:8946–62. 10.1021/es502342r. 25082801

[10] Yan Z , Du K, Yan Y et al. Respiration-driven methanotrophic growth of diverse marine methanogens. Proc Natl Acad Sci USA 2023;120:e2303179120. 10.1073/pnas.2303179120. 37729205 PMC10523532

[11] Bai YN , Wang XN, Wu J et al. Humic substances as electron acceptors for anaerobic oxidation of methane driven by ANME-2d. Water Res 2019;164:114935. 10.1016/j.watres.2019.114935. 31387057

[12] Xie MY , Zhang XQ, Li SQ et al. Humic substances as electron acceptor for anaerobic oxidation of methane (AOM) and electron shuttle in Mn (IV)-dependent AOM. Sci Total Environ 2024;912:169576. 10.1016/j.scitotenv.2023.169576. 38145665

[13] Thauer RK . Biochemistry of methanogenesis: a tribute to Marjory Stephenson. Microbiol-UK 1998;144:2377–406. 10.1099/00221287-144-9-2377. 9782487

[14] Kappler A , Benz M, Schink B et al. Electron shuttling via humic acids in microbial iron(III) reduction in a freshwater sediment. FEMS Microbiol Eco*l* 2004;47:85–92. 10.1016/S0168-6496(03)00245-9. 19712349

[15] Visser SA . Oxidation-reduction potentials + capillary activities of humic acids. Nature 1964;204:581. 10.1038/204581a0.14238167

[16] Clements AP , Kilpatrick L, Lu WP et al. Characterization of the iron-Sulfur clusters in ferredoxin from acetate-grown Methanosarcina-Thermophila. J Bacteriol 1994;176:2689–93. 10.1128/jb.176.9.2689-2693.1994. 8169218 PMC205409

